# Improving the Reactivity of Sugarcane Bagasse Kraft Lignin by a Combination of Fractionation and Phenolation for Phenol–Formaldehyde Adhesive Applications

**DOI:** 10.3390/polym12081825

**Published:** 2020-08-14

**Authors:** Bin Luo, Zhuan Jia, Hongrui Jiang, Shuangfei Wang, Douyong Min

**Affiliations:** 1 College of Light Industry and Food Engineering, Guangxi University, Nanning 530004, China; luobinrobin123@163.com (B.L.); jiazhuan123@163.com (Z.J.); jianghr1984@163.com (H.J.); wangs@gxu.edu.cn (S.W.); 2 Guangxi Key Laboratory of Clean Pulp & Papermaking and Pollution Control, Guangxi University, Nanning 530004, China

**Keywords:** sugarcane bagasse, kraft lignin, phenolation, phenol–formaldehyde adhesive, plywood

## Abstract

The low reactivity of lignin hinders its application as a phenol substitute in phenol–formaldehyde (PF) resin. Therefore, the combination of fractionation and phenolation was adopted to enhance the reactivity of lignin for preparing a phenol–formaldehyde resin adhesive. Sugarcane bagasse kraft lignin and its fractions were employed to replace 40 wt% of phenol to prepare a PF adhesive. The fractionation increased the reactivity of lignin, however the as-prepared lignin-based PF (LPF) hardly met its application requirements as an adhesive. Therefore, the phenolation of lignin under an acidic condition was adopted to further improve its reactivity. The phenolated lignin was characterized by FTIR, gel permeation chromatography, and NMR, indicating its active sites increased while its molecular weight decreased. The phenolated lignin was used to replace 40 wt% of phenol to prepare a PF adhesive (PLPF) which was further employed to prepare plywood. The results indicated that the combination of fractionation and phenolation effectively enhanced the reactivity of lignin, and eventually improved the properties of the PLPF and its corresponding plywood. The free formaldehyde content of PLPF decreased to 0.16%. The wet bonding strength of the as-prepared plywood increased to 1.36 MPa, while the emission of formaldehyde decreased to 0.31 mL/L.

## 1. Introduction

Wood adhesive is one of the key materials in the manufacture of particleboard, fiberboard and plywood. Phenol–formaldehyde (PF) resin adhesive has been widely used in the production of wood-based panels due to its excellent performance and mechanical properties, stability, chemical durability and flame retardancy [1]. A traditional PF adhesive is prepared from non-renewable petroleum products. The growing shortage and rising price of fossil resources have prompted people to develop renewable and cheap bio-based adhesives. A large number of industrial lignin as a by-product has been produced by pulp and papermaking mill. Since lignin contains a structural unit which is similar to phenol, it can be used to replace or partially replace phenol to produce lignin-based phenol–formaldehyde (LPF) resin [2]. However, industrial lignin has a complicated composition, heterogeneous features and a low reactivity with formaldehyde, resulting in a decreased number of reactive functional groups in the resin, thereby inhibiting curing and lowering the crosslinking density [3]. To date, the fractionation methodologies, including solvent extraction [4,5], selective precipitation at reduced pH values [6,7], and membrane ultrafiltration [8], have been used to improve the homogeneity of lignin which is beneficial for the synthesis of LPF resin. Further activation modifications are required to increase the number of active sites. If lignin can replace phenol in the preparation of LPF after the fractionation, the cost of chemical modification will be eliminated. Otherwise, it is necessary to further activate lignin. There have been many reports on improving the reactivity of lignin through chemical modification, such as phenolation [9,10], hydroxylation [11,12], demethylation [13,14], and graft modification [15]. Among these methods, phenolation has proved to be one of the promising methods to improve lignin’s reactivity. Du et al. [16] reported that the phenolic hydroxyl content of industrial softwood kraft lignin was significantly increased after phenolation. Podschun et al. [17] reported that the phenolic hydroxyl content of organosolv lignin also increased after the phenolation. The phenol was introduced into lignin at not only the C_α_ position, but also the C_γ_ position based on ^31^P NMR analysis. Zhang et al. [18] accomplished the phenolation of de-polymerized hydrolysis lignin, and replaced phenol to prepare LPF resin which had the advantages of a higher storage modulus and good storage stability.

In this study, the fractionation and the phenolation was sequentially applied to enhance the reactivity of sugarcane bagasse kraft lignin. Sugarcane bagasse lignin and its fractions were used directly to prepare the lignin-based PF adhesive as the control. Then, kraft lignin (KL) and its fractions were phenolated and applied to prepare the phenolated lignin-based PF adhesive. The phenolated lignin was characterized by FTIR, ^31^P NMR, ^1^H-^13^C 2D heteronuclear singular quantum correlation (HSQC) NMR, and gel permeation chromatography (GPC). The properties of LPF and PLPF were analyzed according to the National Standard of China (GB/T 14074-2006). The performances of the corresponding plywoods were also analyzed according to the National Standard of China (GB/T 17657-2013). The results indicated that the reactivity of lignin, the properties of the PF adhesive, and the performances of the plywood were significantly enhanced by the combination of fractionation and phenolation.

## 2. Materials and Methods

### 2.1. Material

Phenol, sulfuric acid (98 wt%), 1, 4-dioxane, diethyl ether, formaldehyde solution (37%) and NaOH were purchased from Nanning Lantian Chemical Company (Nanning, China). The *Eucalyptus robusta Smith* veneer was kindly supplied by Nanning Science Sky Technology Co. Ltd. (Nanning, China).

The fractionation of sugarcane bagasse kraft lignin by the sequential solvent extraction have been described in our previous report [19]. Briefly, KL was dissolved in the methanol–acetone mixture. Then, the solution was sequentially precipitated in ethyl acetate, acetate/petroleum ether, and ether. As the result, four technical lignin samples, including KL and three fractions (F1, F2, and F3), were achieved and characterized. The active site value of KL, F1, F2, and F3 was calculated as 1.00, 0.61, 0.69, and 1.29 mmol g^−1^, respectively [20]. The molecular weights (Mw) of KL, F1, F2, and F3 were 3.2 × 10^3^, 4.2 × 10^3^, 3.2 × 10^3^, and 1.9 × 10^3^ g mol^−1^, respectively.

### 2.2. Phenolation of Lignin

Lignin was phenolated according to the reported method [21]. A total of 4 g of phenol was added into a round bottom flask. Then, the temperature was increased to 60 °C to liquefy the phenol. After that, 1 g of the lignin sample was added and suspended in liquefied phenol. The phenolation was completed at 110 °C for 2.5 h with magnetic stirring. When the phenolation was finished, the mixture was quickly cooled to room temperature by running water. The mixture was dissolved in 15 mL of 1, 4-dioxane and then precipitated into 150 mL of diethyl ether. The precipitate was collected by centrifugation and washed with diethyl ether several times to remove excess phenol. The phenolated lignin (PL) was obtained after vacuum drying at 40 °C for 24 h.

### 2.3. Preparation of Adhesive

The phenol–formaldehyde resin (PF) and the lignin-based phenol–formaldehyde resin (LPF) were prepared according to the reported method [20].

#### 2.3.1. PF Adhesives Preparation

Technically, 11.64 g of phenol, 10.14 g of formaldehyde solution (37 wt%) and 0.58 g of NaOH were mixed in a three necked round bottom flask with mechanical stirring. The mixture was heated to 80 °C and maintained for 1 h. Then, the second part of the mixture, composed of 10.14 g of formaldehyde solution (37 wt%) and 0.29 g of NaOH, was added. Then, the suspension was maintained at 80 °C for 1.5 h. As the temperature decreased to 65 °C, an additional 0.29 g of NaOH was added and the reaction was completed at 65 °C for 0.5 h.

#### 2.3.2. LPF Adhesives Preparation

The lignin phenol–formaldehyde (LPF) adhesive was prepared by completing the following procedure. Specifically, 4.66 g of lignin and 6.98 g of phenol were mixed in a flask which was continuously stirred at 40 °C for 0.5 h to obtain the homogenous lignin phenol (LP) adduct. Then, the LP, replacing 40% of phenol, was applied to synthesize the LPF according to the procedure which was described in Section 2.3.1.

### 2.4. Plywood Preparation

A *Eucalyptus robusta Smith* veneer (300 mm × 300 mm × 1.5 mm) was used to prepare a three-layer plywood. The adhesive mixture was prepared by 11.52 g of the as-prepared adhesive and 2.88 g of wheat flour, and the loading of the adhesive mixture was 50 g/m^2^. The coated veneers were hot-pressed at 150 °C under 1.5 MPa for 6 min. The wet bonding strength and the formaldehyde release of plywood were analyzed according to the National Standard of China (GB/T 17657-2013).

### 2.5. Characterization

#### 2.5.1. Fourier Transform Infrared Spectrometer Analysis (FT-IR)

A FT-IR analysis of the sample was performed on a BRUKER TENSOR II instrument (Bruker, Karlsruhe, Germany). A total of 1 mg of the sample mixed with 200 mg of potassium bromide (KBr) was pressurized to achieve a transparent pallet. Each spectrum was recorded in a frequency range of 600–4000 cm^−1^ and scanned 16 times with a resolution of 4 cm^−1^.

#### 2.5.2. GPC Analysis

KL and phenolated kraft lignin (PKL) were respectively acetylated according to the reported method [22]. The molecular weight of the acetylated sample was determined using a 1200 gel permeation chromatograph (Agilent, Santa Clara, CA, USA). Tetrahydrofuran was used as the mobile phase. The column temperature was 30 °C, and the flow rate was 0.7 mL/min. The sample concentration was 1 mg/mL and the injection volume was 20 μL. An Agilent PL gel MIXED-E column (7.5 × 300 mm, 3 μm) ranging from 500 to 3 × 10^4^ was employed. Polystyrene of different molecular weights (Mw = 500, 2 × 10^3^, 5 × 10^3^, 1 × 10^4^, and 3 × 10^4^ g/mol, polydispersity index (PDI) < 1.20) was applied for the standard curve. Each test was replicated three times and the average values were reported.

#### 2.5.3. ^1^H-^13^C HSQC NMR Analysis

A ^1^H-^13^C HSQC NMR analysis of the sample was conducted in a Bruker Avance 500 MHz NMR (Bruker, Germany). Briefly, 150 mg of the sample was dissolved in 500 μL of dimethyl sulfoxide (DMSO-*d*_6_) and transferred into an NMR tube. The acquisition parameters were as follows: F1 spectral width (^1^H), 19.9947 Hz; scanning number, 16; pulse width selection of P1, 12 μs; acquisition point (TD), 65536; F2 spectral width (^13^C), 12.9836 Hz; scanning number, 10,000; pulse width of P1, 12 μs; acquisition point (TD), 1024. The chemical shift was corrected according to the DMSO contour (δ_C_/δ_H_ 39.5/2.5 ppm).

#### 2.5.4. ^31^P NMR Analysis

A ^31^P NMR analysis of the sample was also conducted in a Bruker Avance 500 MHz NMR (Bruker, Germany). ^31^P NMR spectra were acquired according to the reported methods [23,24,25]. Briefly, 20 mg of the sample was completely dissolved in 500 μL of anhydrous pyridine and deuterated chloroform (1.6:1, *v*/*v*). Then, 100 μL of cyclohexanol (10.85 mg/mL) and 100 μL of chromium (III) acetylacetonate solution (5 mg/mL in pyridine and deuterated chloroform 1.6:1, *v*/*v*) were added as the internal standard and the relaxation reagent, respectively. Subsequently, 100 μL of phosphitylating reagent (2-chloro-4,4,5,5-tetramethyl-1,3,2-dioxaphosphol) was added. The reaction was completed for 15 min under stirring. The product was transferred into an NMR tube for analysis. All chemical shifts were corrected to the reaction product (2-chloro-4, 4,5,5-tetramethyl-1,3,2-dioxaphosphol) which gave a sharp signal at 132.2 ppm in pyridine/CDCl_3_.

#### 2.5.5. Properties of Adhesive

The adhesive properties, including the solid content, free phenol content, and free formaldehyde content, were determined according to the National Standard of China (GB/T 14074-2006). Each test was replicated three times and the average values were reported.

#### 2.5.6. Properties of Plywood

**Figure 1:**
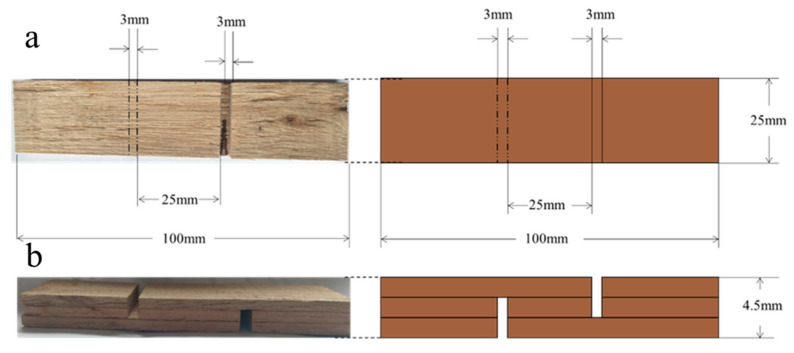
Specimen used for the bonding strength testing: (**a**) top view, (**b**) side view.

The wet bonding strength of plywood was tested according to the procedures specified by the National Standard of China (GB/T 14074-2006). The formaldehyde release was determined through the desiccator method described by the National Standard of China (GB/T 17657-2013). The specs of the test piece are shown in Figure 1. An electronic universal testing machine (Instron Corporation, Norwood, MA, USA) and a plate curing instrument (Jinrunqi Rubber Machinery Co., Ltd., Qingdao, China) were used. The wet bonding strength of the plywood specimen was tested as follows. The specimen was boiled in water for 4 h. Then the specimen was dried at 63 °C for 20 h. The specimen was boiled in water for another 4 h. After 10 min cooling, the specimen was used for test. The tensile strength of specimen was measured at a constant loading speed of 10 MPa/min. The maximum load was recorded to 10 N accuracy. Each test was replicated three times and the average values were reported.

## 3. Results and Discussion

### 3.1. Adhesive Properties and Plywood Performance

The properties of the LPF adhesive and its plywood performance are summarized in Table 1. LPF possessed higher a solid content than PF because of its specific colloidal properties [26]. Meanwhile, LPF emitted massive free formaldehyde (approximately 1%), exceeding the National Standard of China (GB/T14732-2006) (≤0.30%). This can be explained by the low reactivity of lignin. Although the bonding strength of plywood prepared with LPF exceeded the plywood (Class I) national requirements of China (≥0.70 MPa), it was significantly lower than the counterpart prepared with PF (1.49 MPa). On the other hand, the formaldehyde release of plywood prepared with LPF (1.09 mg/L~1.21 mg/L) exceeded the National standard of China (GB/T 9846.3-2004) (≤0.50 mg/L). Table 1 shows that the fractionation of lignin enhanced the adhesive properties and plywood performance through the improvement of the homogeneity and reactivity of lignin. Compared to KL, for instance, LPF prepared from its fractions possessed less free formaldehyde and free phenol (<1.46%), but a higher bonding strength (>0.68 MPa), and its plywood emitted less formaldehyde (<1.21 mg/L). However, the improvement hardly assured that lignin and its fractions were suitable to replace phenol for preparing PF adhesives. Therefore, the phenolation is the prerequisite step to improve the reactivity of lignin.

### 3.2. Characterization of Phenolated Lignin

The lignin sample was phenolated under the acidic condition which was subsequently characterized by FTIR, GPC, ^1^H-^13^C HSQC NMR, and ^31^P NMR.

#### 3.2.1. FT-IR Analysis

**Figure 2:**
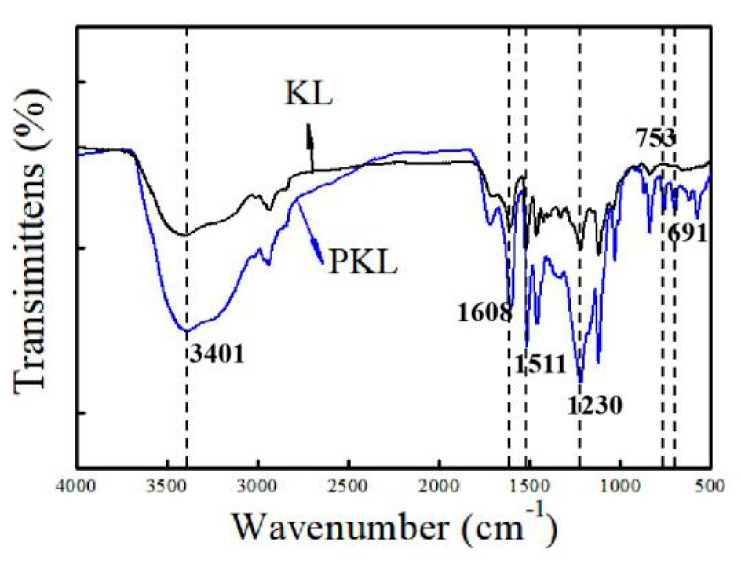
FT-IR spectra of KL and PKL.

The FT-IR spectra of KL and PKL were demonstrated in Figure 2. The main peaks of the samples were assigned according to the reported literature [26,27]. The enhancement of the intensity of the peak at 3401 cm^−1^, assigned to the stretching vibration frequency of O–H (Figure 2), revealed the increase in the phenolic hydroxyl group after phenolation. The peaks at 1608 and 1511 cm^−1^ were assigned to aromatic ring vibrations. The increasing intensity of the band at 1230 cm^−1^, assigned to the bending vibration of the phenolic hydroxyl group (O–H), indicated that lignin was successfully phenolated (Figure 2). Besides, the emerging characteristic peaks at 753 and 691 cm^−1^ assigned to the para position substitution of phenol confirmed the phenolation of lignin (Figure 2) [27].

#### 3.2.2. GPC Analysis

Table 2 demonstrates the changes of the molecular weight and PDI of KL and PKL after the phenolation which was consistent with the reported result [10]. Generally, the molecular weight and polydispersity of lignin were decreased after phenolation. For example, the average molecular weight (Mw) decreased from 3.2 × 10^3^ to 1.9 × 10^3^ g/mol, and the PDI decreased from 3.01 to 2.13. The decrease in molecular weight combining with the increase in phenolic hydroxyl indicated the reactivity improvement of lignin. Meanwhile, the decrease in the PDI revealed phenolation also improved the homogeneity of lignin. Conclusively, the improvement of lignin properties after phenolation were beneficial to apply the phenolated lignin as the raw material for the development of the downstream products.

#### 3.2.3. ^1^H-^13^C HSQC NMR Analysis

**Figure 3:**
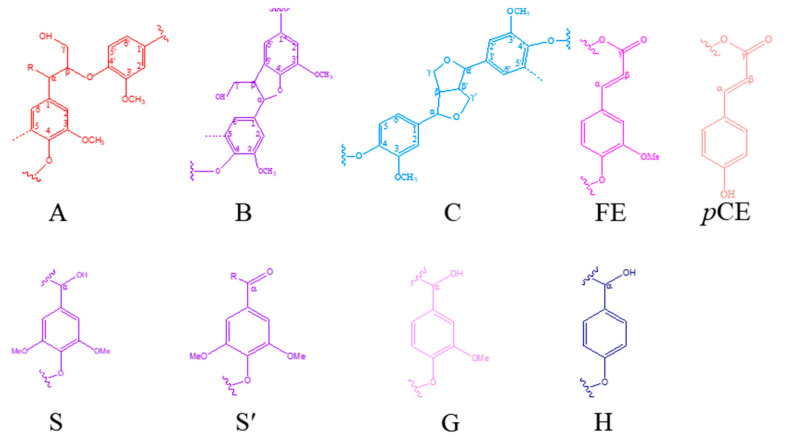
Main interunit linkages and aromatic units of lignin: (**A**) β-O-4′, (**B**) β-5′, (**C**) β-β′, (**FE**) ferulate ester, (***p*CE**) *p*-coumarate ester, (**S**) syringyl units, (**S′**) Cα-oxidized syringyl unit, (**G**) guaiacyl unit, (**H**) *p*-hydroxyphenyl unit.

The main substructures of lignin identified by 2D NMR are shown in Figure 3.

The structural changes of lignin after phenolation were characterized by ^1^H-^13^C HSQC NMR (Figure 3). Technically, the 2D NMR spectrum was defined as two regions: the oxygenated side region (δ_C_/δ_H_ 50–90/2.5–6.5 ppm) and aromatic region (δ_C_/δ_H_ 100–140/5.5–8.0 ppm).

**Figure 4:**
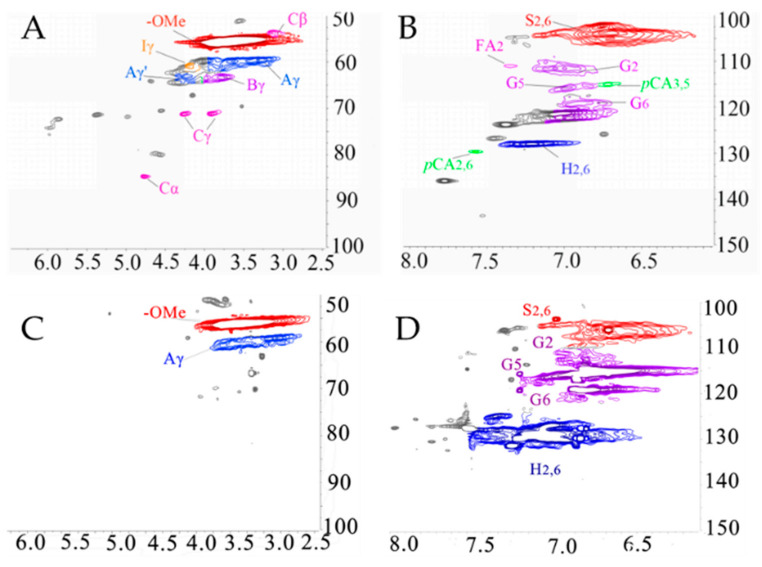
2D HSQC NMR spectra: (**A**) oxygenated side region of KL, (**B**) aromatic region of KL, (**C**) oxygenated side region of PKL, (**D**) aromatic region of PKL.

The main peaks of the lignin samples were assigned according to the reported literature [19,28]. For example, the peak at δC/δH 53.3/3.48 ppm was attributed to the C_β_-H_β_ in β-5′ substructures (**B**). The peak at δC/δH 53.5/3.08 ppm was attributed to the C_β_-H_β_ in pinoresinol substructures (**C**). The peak at δC/δH 60.0/3.43 ppm was attributed to the C_γ_-H_γ_ in β-O-4′ substructures (**A**). The peak at δC/δH 63.1/4.36 ppm was attributed to the C_γ_-OH of alkyl-aryl ethers (β-O-4) acylated with *p*CE. The peak at δC/δH 103.8/6.96 ppm was assigned to the C_2,6_-H_2,6_ in syringyl units (**S**). The peak at δC/δH 127.9/7.01 ppm was assigned to the C_2,6_-H_2,6_ in *p*-hydroxyphenyl units (**H**). Meanwhile, it was shown in Figure 4 that the oxygenated side of lignin changed significantly after the phenolation [10]. For example, C_α_-OH was converted into a carbon cation under the acidic condition. Then, the carbon cation linked to the ortho/para-position of phenol through an electrophilic reaction which induced the increase in phenolic hydroxyl groups and the decrease in aliphatic hydroxyl groups. Meanwhile, the β-elimination of the γ-hydroxymethyl group as a formaldehyde under the acidic condition was completed. The released formaldehyde reacted with phenol and lignin fractions to form diphenylmethanes [10]. Compared to KL, the intensity of the signal at δ_C_/δ_H_ 130/7.0 ppm assigned to the H_2,6_ in PKL was significantly increased indicating the success of phenol introduced onto lignin molecules. Meanwhile, the signal attributed to S_2,6_ shifted from δ_C_/δ_H_ 103.8/6.69 ppm to 105.1/6.52 ppm after phenolation, confirming that the phenolation reaction was completed at the side chain of lignin [10].

#### 3.2.4. ^31^P NMR Analysis

**Figure 5:**
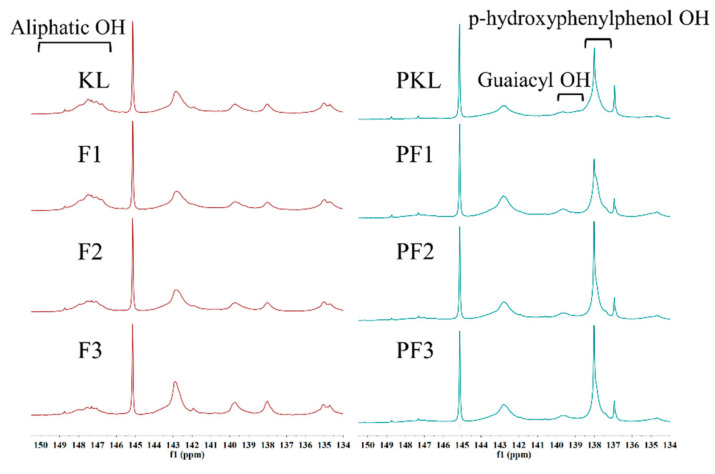
^31^P NMR spectrum of lignin and its corresponding phenolated sample.

The ^31^P NMR spectra of the samples are shown in Figure 5, and the quantitative analyses are summarized in Table 3. The integral region of H–OH was 138.5~136.9 ppm. The integral region of the G–OH was 140.2~138.5 ppm. Figure 5 shows that lignin contains non-condensed guaiacyl OH (G–OH) and *p*-hydroxyphenylphenol OH (H–OH) which contains one and two active sites for the synthesis of phenolic aldehyde resin adhesives, respectively [21]. After the phenolation, the intensity of the signal at 138.1 ppm assigned to H–OH was significantly enhanced (Figure 5), indicating the increase in *p*-hydroxyphenyl units due to the introduction of phenol on lignin. In addition, the non-condensed G-OH was increased if phenol was linked to the Cα of lignin through the ortho position. In contrast, the phenolated lignin possessed a relatively weak signal at 147.5 ppm assigned to the aliphatic hydroxyl group. The non-condensed H–OH was increased if phenol was linked to the Cα of lignin through the para position. The Cα of KL was mainly linked with –OH or –OR. Therefore, the para position-substituted phenol was determined as H–OH, and the ortho-substituted phenol was determined as G–OH. Here, the content of Cα-OH at 146–150 ppm contributes to the total content of aliphatic OH. When sulfuric acid was employed as the catalyst, the groups at this position were easily replaced by phenol during phenolation [29]. The decreased aliphatic OH content of the phenolated lignin under acidic conditions was consistent with the reported result [10]. The ^31^P NMR analysis indicated that the phenolated lignin contains more Ph–OH groups and free reactive sites, thereby improving its reactivity towards thermoset applications as a phenol substitute. The improvement of the reactivity is beneficial to improve the adhesive strength and storage stability of LPF [18].

Conclusively, lignin was successfully phenolated under the acidic condition. After the phenolation, the content of Ph–OH was increased significantly which enhanced the reactivity of lignin through the improvement of active sites. For example, the active sites of F1, F2 and F3 after the phenolation were 9.8, 8.5, and 5.3 times higher, respectively, than the corresponding original samples. The largest amount of the active sites was quantified from PF3 which was 6.86 mmol/g. Therefore, PF3 was the most suitable candidate to replace phenol for the synthesis of LPF resin.

### 3.3. PLPF Adhesive Properties and Plywood Performances

The phenolated KL and its fractions were applied to replace 40% of phenol to prepare PF adhesives which were nominated as PKLPF, PF1PF, PF2PF, and PF3PF. The phenolated lignin-based PF adhesive’s properties, as well as the bonding strength and the formaldehyde release of the corresponding plywood, are demonstrated in Table 1.

The free formaldehyde content of the phenolated lignin-based adhesive was significantly lower than the counterpart of the lignin-based adhesive. For example, the free formaldehyde decreased from 1.04% (KLPF, in Table 1) to 0.19% (PKLPF), which was lower than the limitation requirement of the China National Standard (≤0.3%). The reduction in the free formaldehyde, a key factor of PF adhesives, was explained by the enhancement of the reactivity and homogeneity of lignin induced by the phenolation. Consequently, the wet bonding strength of the corresponding plywood was significantly increased while its formaldehyde release decreased. After the phenolation, the wet bonding strength of plywood prepared increased from 0.68 MPa (KLPF as the adhesive) to 1.27 MPa (PKLPF as the adhesive) as an example. The highest wet bonding strength of plywood was achieved as 1.36 MPa when PF3FP was used. Furthermore, the formaldehyde release of the plywood was as significantly reduced when the phenolated lignin-based adhesive was employed. It was found that the formaldehyde release decreased from 1.21 mg·L^−1^ (KLPF as the adhesive) to 0.33 mg·L^−1^ (PKLPF as the adhesive). The lowest formaldehyde release of the plywood prepared with PF3PF was only 0.31 mg·L^−1^ which is significantly lower than the limitation requirement of the China National Standard (GB/T 9846.3-2004). Conclusively, phenolation is an effective and facile method to improve the reactivity of lignin and eventually enhance the properties of a lignin-based adhesive and its plywood.

## 4. Conclusions

In the current study, the combination of fractionation and phenolation was proposed to facilitate the application of lignin for preparing phenol–formaldehyde resin. Lignin and its fractions were phenolated and characterized. The result revealed that the fractionation improved the reactivity and homogeneity of lignin, however the properties of LPF and the performances of the corresponding plywood were little able to meet the China National Standard requirement limitations. Therefore, the phenolation was sequentially adopted to further improve the reactivity of lignin and its fractions. The result indicated that the plywood prepared from the phenolated lignin-based PF adhesive possessed higher wet bonding strengths, and a lower formaldehyde release. It was illustrated that the combination of the fractionation and phenolation can effectively enhance the reactivity of lignin, and eventually improve the properties of a PLPF and its corresponding plywood. Therefore, the proposed route offers a new promising alternative approach for preparing phenolated lignin with a good potential for application in phenol–formaldehyde resin adhesives.

## Figures and Tables

**Table 1:** The Properties of Lignin and Phenolated Lignin-Based Adhesive and the Performances of Plywood.

Sample	Adhesive Properties				Plywood Performance	
	pH	Solid Content (%)	Free Formaldehyde (%)	Free Phenol (%)	Wet Bonding Strength (MPa)	Formaldehyde Release (mg/L)
PF	11.9 ± 0.1	38.1 ± 0.6	0.12 ± 0.02	0.24 ± 0.03	1.49 ± 0.06	0.19 ± 0.03
KLPF	11.2 ± 0.2	42.1 ± 0.5	1.04 ± 0.05	0.72 ± 0.01	0.68 ± 0.11	1.21 ± 0.07
F1-LPF	11.2 ± 0.1	42.3 ± 0.7	1.18 ± 0.07	0.65 ± 0.08	0.98 ± 0.17	1.18 ± 0.02
F2-LPF	11.2 ± 0.2	43.3 ± 0.8	0.91 ± 0.11	0.44 ± 0.04	0.83 ± 0.08	1.12 ± 0.03
F3-LPF	11.3 ± 0.1	45.8 ± 0.5	0.98 ± 0.05	0.47 ± 0.01	0.93 ± 0.12	1.09 ± 0.05
PKLPF	11.5 ± 0.1	42.8 ± 0.8	0.19 ± 0.12	1.33 ± 0.03	1.27 ± 0.09	0.33 ± 0.06
PF1PF	11.4 ± 0.2	44.1 ± 0.7	0.25 ± 0.08	1.59 ± 0.07	1.19 ± 0.16	0.39 ± 0.07
PF2PF	11.4 ± 0.1	43.9 ± 0.5	0.27 ± 0.04	0.92 ± 0.02	1.15 ± 0.13	0.43 ± 0.05
PF3PF	11.9 ± 0.1	44.6 ± 0.7	0.16 ± 0.05	1.46 ± 0.02	1.36 ± 0.11	0.31 ± 0.06
GB/T14732	≥7.0	≥35.0	≤0.30	≤6.00	≥0.70	≤0.50 ^a^

Note: value ^a^ was cited from GB/T 9846.3-2004.

**Table 2:** GPC Analysis of KL and PKL.

Sample	Mw (g/mol)	PDI
KL	3.2 × 10^3^	3.01
PKL	1.9 × 10^3^	2.13

**Table 3:** Quantitative ^31^P NMR Analyses of Lignin and its Corresponding Phenolated Sample.

Sample	–OH (mmol/g)								
	S	G	NC–OH ^a^			–COOH	Aliphatic–OH	Total –OH	Active Sites ^b^
			S	G	H				
KL	0.01	0.22	1.23	0.35	0.15	0.38	0.71	1.96	0.65
PKL	0.08	0.30	0.93	0.61	3.01	0.15	0.27	4.93	6.63
F1	0.01	0.28	1.02	0.33	0.14	0.38	0.92	1.78	0.62
PF1	0.09	0.43	0.94	0.55	2.77	0.25	0.13	4.78	6.08
F2	0.02	0.24	1.37	0.37	0.16	0.34	0.44	2.16	0.69
PF2	0.11	0.44	1.10	0.62	2.63	0.18	0.29	4.90	5.88
F3	0.07	0.18	1.73	0.53	0.38	0.45	0.28	2.89	1.29
PF3	0.08	0.30	1.24	0.61	3.12	0.11	0.67	5.35	6.86

Note: NC–OH ^a^ stands for non-condensed hydroxyl group. Active sites ^b^ is calculated as the following equation, active sites = G–OH + 2 × H–OH.

## References

[1] Lee S.H., Yoshioka M., Shiraishi N. (2000). Preparation and properties of phenolated corn bran (CB)/phenol/formaldehyde cocondensed resin. J. Appl. Polym. Sci..

[2] Sjöström E. (1981). Wood Chemistry: Fundamentals and Applications.

[3] Falkehag S.I., Braddon D.V., Dougherty W.K. (1975). Lignin polymer applications. Renewable Resources for Plastics: Growth and Change in Adhesives, Chemical Requirements of the Tobacco Industry.

[4] Yang S., Wen J.L., Yuan T.Q., Sun R.C. (2014). Characterization and phenolation of biorefinery technical lignin for lignin-phenol-formaldehyde resin adhesive synthesis. RSC Adv..

[5] Li M.F., Sun S.N., Xu F., Sun R.C. (2012). Sequential solvent fractionation of heterogeneous bamboo organosolv lignin for value-added application. Sep. Purif. Technol..

[6] Cui C., Sun R., Argyropoulos D.S. (2014). Fractional Precipitation of Softwood Kraft Lignin: Isolation of Narrow Fractions Common to a Variety of Lignins. ACS Sustain. Chem. Eng..

[7] dos Santos P.S., Erdocia X., Gatto D.A., Labidi J. (2014). Characterisation of Kraft lignin separated by gradient acid precipitation. Ind. Crops Prod..

[8] Wang G., Chen H. (2013). Fractionation of alkali-extracted lignin from steam-exploded stalk by gradient acid precipitation. Sep. Purif. Technol..

[9] Jamin M., Adam M., Damblon C., Christiaens L., Frère J.M. (1991). Lignin separation and fractionation by ultrafiltration. Biochem. J..

[10] Jiang X., Liu J., Du X., Hu Z., Chang H., Jameel H. (2018). Phenolation to improve lignin reactivity towards thermosets application. ACS Sustain. Chem. Eng..

[11] Hong S., Lian H., Sun X., Pan D., Carranza A., Pojman J.A., Mota-Morales J.D. (2016). Zinc-based deep eutectic solvent-mediated hydroxylation and demethoxylation of lignin for the production of wood adhesive. RSC Adv..

[12] Zhou W., Chen F., Zhang H., Wang J. (2017). Preparation of a polyhydric aminated lignin and its use in the preparation of polyurethane film. J. Wood Chem. Technol..

[13] Nagieb Z.A. (1985). Demethylation of thiolignin by reaction with potassium dichromate-a kinetic study. Wood Sci. Technol..

[14] Xia C.L., Xu Y.Z., Liu X.H., Wang C.P. (2016). Preparation and characterization of demethylated lignin assisted by microwave irradiation. Chem. Ind. For. Prod..

[15] Chile L., Kaser S.J., Hatzikiriakos S.G., Mehrkhodavandi P. (2018). Synthesis and Thermorheological Analysis of Biobased Lignin-graft-poly (lactide) Copolymers and Their Blends. ACS Sustain. Chem. Eng..

[16] Du X., Li J., Lindström M.E. (2014). Modification of industrial softwood kraft lignin using Mannich reaction with and without phenolation pretreatment. Ind. Crops Prod..

[17] Podschun J., Stücker A., Saake B., Lehnen R. (2015). Structure–Function Relationships in the Phenolation of Lignins from Different Sources. ACS Sustain. Chem. Eng..

[18] Zhang Y., Yuanauthor Z., Xuauthor C. (2016). Sustainable bio-phenol-hydroxymethylfurfural resins using phenolated de-polymerized hydrolysis lignin and their application in bio-composites. Ind. Crops Prod..

[19] Jia Z., Li M., Wan G., Luo B., Guo C., Wang S., Min D. (2018). Improving the homogeneity of sugarcane bagasse kraft lignin through sequential solvents. RSC Adv..

[20] Yang S., Wu J., Zhang Y., Yuan T., Sun R. (2015). Preparation of lignin-phenol-formaldehyde resin adhesive based on active sites of technical lignin. J. Biobased Mater. Bio..

[21] Aonso M.V., Oliet M., Rodrıguez F., Garcıa J., Gilarranz M.A., Rodr Guez J.J. (2005). Modification of ammonium lignosulfonate by phenolation for use in phenolic resins. Bioresour. Technol..

[22] Glasser W.G., Davé V., Frazier C.E. (1993). Molecular weight distribution of (semi-) commercial lignin derivatives. J. Wood Chem. Technol..

[23] Argyropoulos D.S. (1994). Dimitris quantitative phosphorus-31 NMR analysis of six soluble lignin. J. Wood Chem. Technol..

[24] Granata A., Argyropoulos D.S. (1995). 2-Chloro-4,4,5,5-tetramethyl-1,3,2-dioxaphospholane, a reagent for the accurate determination of the uncondensed and condensed phenolic moieties in lignins. J. Agric. Food Chem..

[25] Guerra A., Filpponen I., Lucia L.A., Saquing C., Baumberger S., Argyropoulos D.S. (2006). Toward a better understanding of the lignin isolation process from wood. J. Agric. Food Chem..

[26] Jia Z., Wan G., Zhang Q., Li M., Luo B., Guo C., Wang S., Min D. (2018). Phenolation of bagasse kraft lignin for application in lignin-based phenol formaldehyde adhesives. China Pulp. Paper.

[27] Li C., Wang W., Mu Y., Zhang J., Zhang S., Li J., Zhang W. (2017). Structural properties and copolycondensation mechanism of valonea tannin-modified phenol-formaldehyde resin. J. Polym. Environ..

[28] Zhang Q., Wan G., Li M., Jiang H., Wang S., Min D. (2020). Impact of bagasse lignin-carbohydrate complexes structural changes on cellulase adsorption behavior. Int. J. Biol. Macromol..

[29] Song Y., Wang Z., Yan N., Zhang R., Li J. (2016). Demethylation of wheat straw alkali lignin for application in phenol formaldehyde adhesives. Polymers.

